# *Plasmodium vivax* Malaria in Cambodia

**DOI:** 10.4269/ajtmh.16-0208

**Published:** 2016-12-28

**Authors:** Sovannaroth Siv, Arantxa Roca-Feltrer, Seshu Babu Vinjamuri, Denis Mey Bouth, Dysoley Lek, Mohammad Abdur Rashid, Ngau Peng By, Jean Popovici, Rekol Huy, Didier Menard

**Affiliations:** 1 National Center for Parasitology, Entomology and Malaria Control (CNM), Phnom Penh, Cambodia.; 2 Malaria Consortium Cambodia, Phnom Penh, Cambodia.; 3 World Health Organization, Country Office, Phnom Penh, Cambodia.; 4 Institute Pasteur in Cambodia (IPC), Phnom Penh, Cambodia.

## Abstract

The Cambodian National Strategic Plan for Elimination of Malaria aims to move step by step toward elimination of malaria across Cambodia with an initial focus on *Plasmodium falciparum* malaria before achieving elimination of all forms of malaria, including *Plasmodium vivax* in 2025. The emergence of artemisinin-resistant *P. falciparum* in western Cambodia over the last decade has drawn global attention to support the ultimate goal of *P. falciparum* elimination, whereas the control of *P. vivax* lags much behind, making the 2025 target gradually less achievable unless greater attention is given to *P. vivax* elimination in the country. The following review presents in detail the past and current situation regarding *P. vivax* malaria, activities of the National Malaria Control Program, and interventional measures applied. Constraints and obstacles that can jeopardize our efforts to eliminate this parasite species are discussed.

## Background

**Figure 1.**
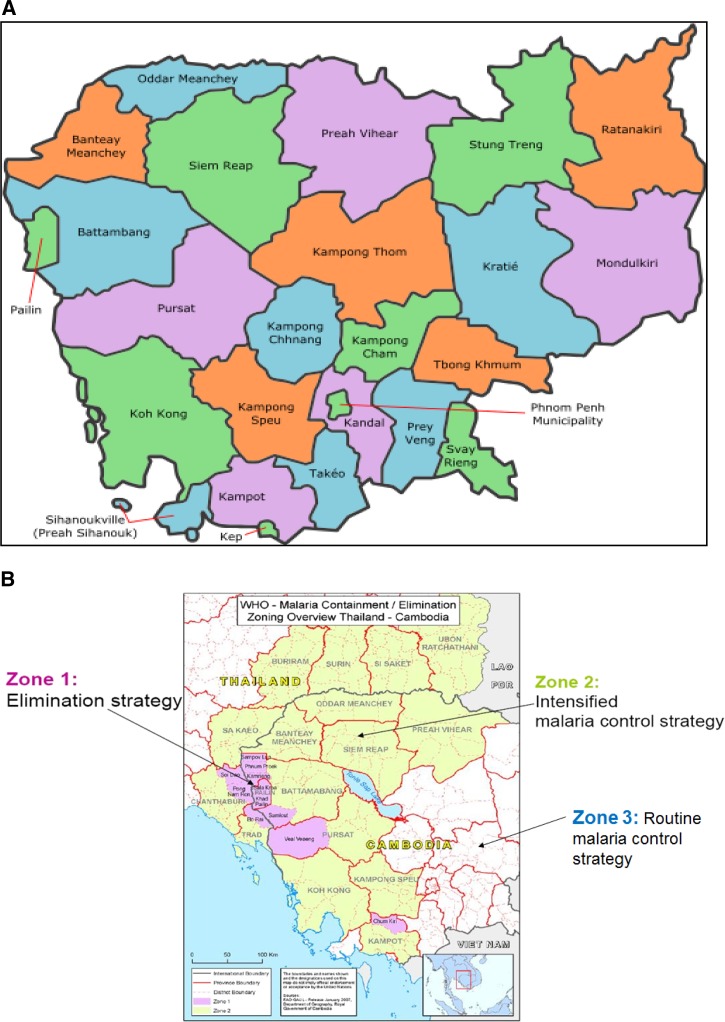
Map of Cambodia showing (**A**) delimitation of the 25 provinces and the Phnom Penh municipality, Cambodia 2015, and (**B**) delimitation of the three zones defined in 2009 for the World Health Organization, malaria containment/elimination strategy, Cambodia.

The Kingdom of Cambodia, which lies in the center of the Indochina peninsula in southeastern Asia has a population of 15,840,251 (in 2015) consisting of 2.2 million households, in 13,406 villages in 25 provinces and one municipality (Phnom Penh) (Figure 1A

). The control of malaria and other vector-borne diseases remains a very high priority for the Ministry of Health, particularly to the National Center for Parasitology, Entomology and Malaria Control (CNM), a specialized institution founded to develop and execute a nationwide malaria control strategy in Cambodia. Despite a substantial decline in its incidence, malaria continues to be a leading public health concern, where both *Plasmodium falciparum* and *Plasmodium vivax* coexist. The emergence of artemisinin-resistant *P. falciparum* in western Cambodia over the last decade has drawn global attention to support its “containment” with an ultimate goal of *P. falciparum* elimination. However, the control of *P. vivax* lags much behind.

The Cambodian National Strategic Plan for Elimination of Malaria,^1^ which was endorsed by the Cambodian Ministry of Health, aims to move toward preelimination of malaria across Cambodia, bringing the incidence of malaria case below two cases per 1,000 population for most parts, with special efforts to contain artemisinin-resistant *P. falciparum* malaria; by 2020, to move toward elimination of malaria across Cambodia with an initial focus on *P. falciparum* malaria and ensure zero deaths from malaria, and by 2025, to achieve phased elimination of all forms of malaria in Cambodia, including *P. vivax* malaria.

The following review presents in detail the past and current situation of the vivax malaria in Cambodia, malaria control activities, and interventional measures currently implemented by the CNM along with relevant recommendations. Major constraints and obstacles which can jeopardize the elimination of *P. vivax* are also discussed.

## Malaria Control in Cambodia: Past and Present

Interventions to reduce malaria in Cambodia started in 1951 as part of the Global Malaria Eradication Campaign. Following a 6-year indoor residual spraying (IRS) campaign using dichlorodiphenyltrichloroethane, malaria prevalence rates reduced from 60% to 1% by the early 1960s.^2^ From 1970 to 1975, malaria activities were disrupted by the civil war. During the period 1975 to 1978, all malaria control activities were discontinued. In 1984, the Cambodian Ministry of Health founded and designated a specialized institution, the CNM, to provide technical and material support to malaria treatment facilities in provincial and district hospitals and to develop and execute a nationwide malaria control strategy. It was not until the early 1990s that logistical support for malaria diagnosis and treatment became integrated with the national public health system and the CNM transitioned from purely hospital-based curative activities to more proactive community-based health education and control activities.

**Figure 2.**
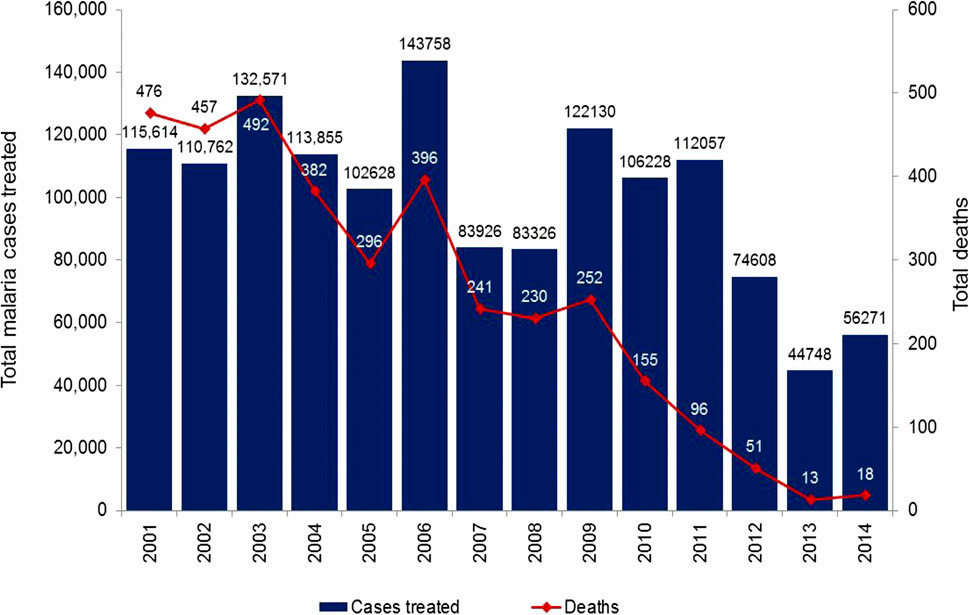
Malaria cases and deaths reported by public facilities and village malaria workers, 2001–2014.

In 2001, the country introduced artemisinin-based combination therapies (ACT)s (artesunate plus mefloquine) at a national scale.^3^ With support from the Global Fund to Fight AIDS, Tuberculosis, and Malaria (GFATM), the CNM successfully extended access to insecticide-treated bed nets and diagnosis and treatment. In 2004, CNM piloted the Village Malaria Worker (VMW) program (community health volunteers trained by the CNM and partners to deliver malaria prevention and treatment services to remote hyperendemic villages), which was expanded in 2009 to all at-risk villages located beyond 5 km from the nearest health facilities.^4^–^6^ After initial confirmation of artemisinin resistance in 2008,^7^,^8^ a containment project was launched in 2009 along the Cambodian–Thai border (in an area defined as Zone 1, see Figure 1B for details) to increase coverage of control interventions, especially vector control (long-lasting impregnated nets [LLINs] and hammock nets) and case management, and limit factors contributing to the spread of resistance.^9^ As observed in the past 10 years in the Greater Mekong Subregion, the number of reported malaria cases has been halved, from 113,855 cases in 2004 to 56,271 cases in 2014. In the same period, the number of malaria deaths has dropped 21-fold (from 382 to 18) (Figure 2

).

## Epidemiological Data on Vivax Malaria in Cambodia

**Figure 3.**
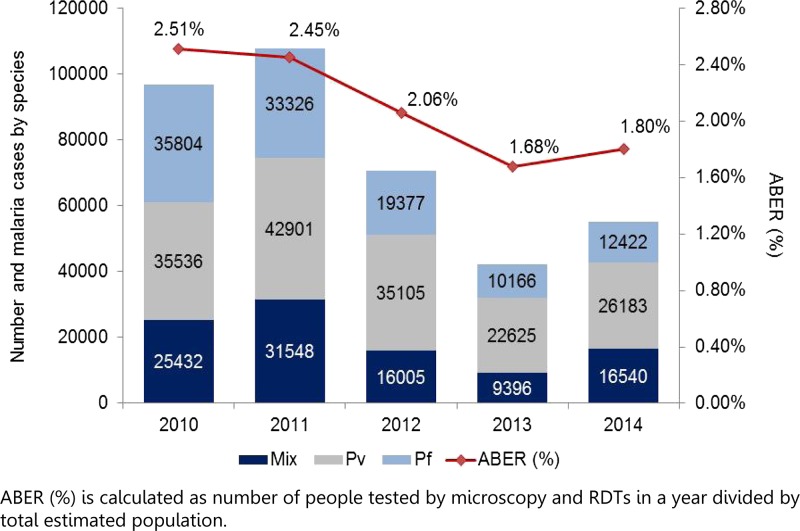
Malaria cases in public sector by species, and annual blood examination rate, 2010–2014.

**Figure 4.**
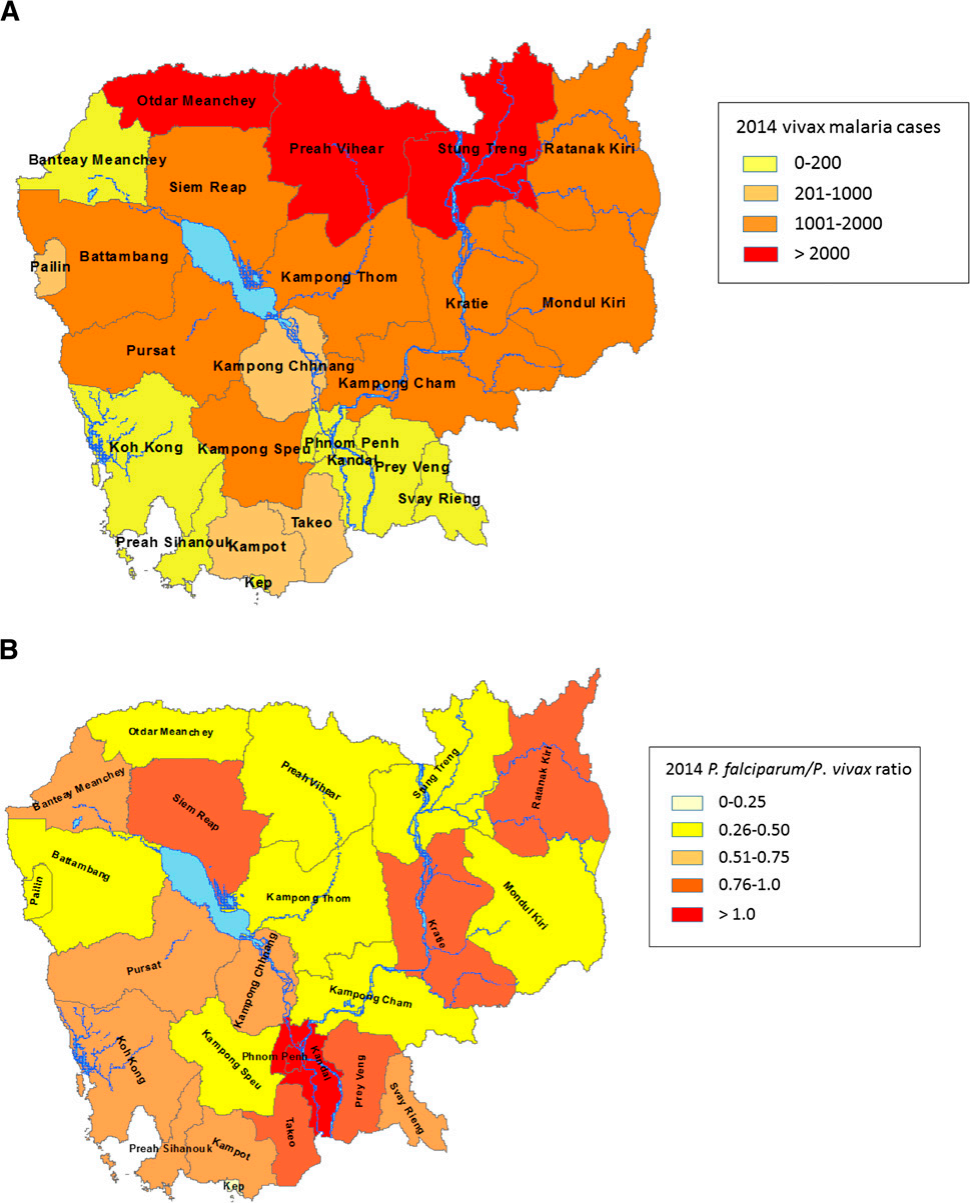
Distribution of *Plasmodium vivax* cases recorded in public sector in 2014, Cambodia. (**A**) Number of confirmed *P. vivax* cases by province and (**B**) ratio of *Plasmodium falciparum*/*P. vivax* cases by province.

*Plasmodium falciparum* was the predominant species among confirmed malaria cases until 2010. With the roll out and scale-up of multispecies (Pan) rapid diagnostic tests (RDTs) in 2009 (before 2009, malaria RDT used detected only *P. falciparum* parasites—HRP2-based detection), the proportion of infections due to *P. falciparum* shifted and for the first time in 2011, the number of *P. vivax* cases (42,901) was higher than the number of *P. falciparum* cases (33,326) registered in the public sector (health centers, provincial hospital, and VMW data). In 2014, based on malaria RDT detection (health centers and VMW data), *non-P. falciparum* infections (mostly *P. vivax*) accounted for 46% (26,183) of cases, followed by 32% (16,540) of mixed infections of both *P. falciparum* and *non-P. falciparum* and 21% (12,422) of pure *P. falciparum* cases (Figure 3

). *Plasmodium vivax* cases are currently largely distributed in six provinces in northeastern Cambodia (20,954, ∼80%) and particularly prevail in Stung Treng Province along the Lao People's Democratic Republic–Cambodia border (∼28% of the total reported vivax cases) (Figure 4

).

**Figure 5.**
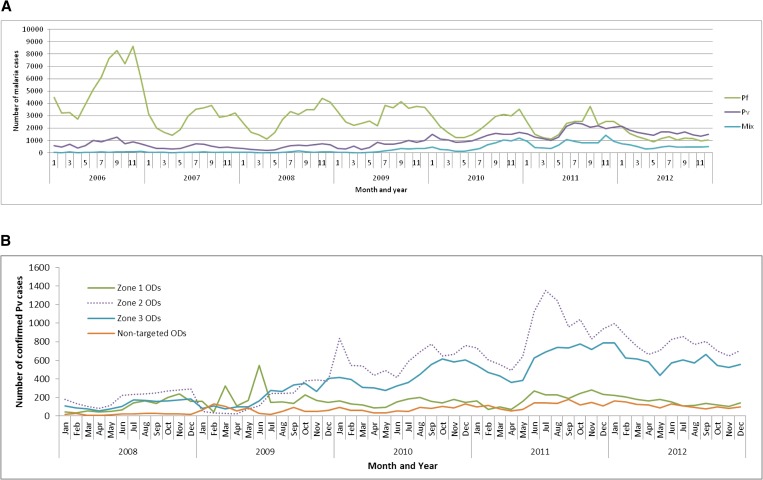
(**A**) Number of malaria cases by species and their seasonal variations between 2006 and 2012, Cambodia. (**B**) Number of malaria cases by zones (the definition of each zone is given in Figure 1, panel B) between 2008 and 2012, Cambodia. OD = operational districts.

Malaria transmission and *P. vivax* cases in Cambodia are perennial over the year, but usually peak between June and November corresponding to the rainy season and with quite a similar pattern to that of *P. falciparum* (Figure 5A

). From 2006 to 2012, there was a jump in the number of *P. vivax* cases between Zone 1, where intense interventional efforts were implemented as part of the artemisinin resistance containment activities from 2009 and the other areas (defined as Zones 2 and 3, see Figure 1B). By comparing the number of *P. vivax* cases from 2009 by zone, the increases were less prominent in Zone 1, where artesunate plus mefloquine regimen was replaced by dihydroartemisinin plus piperaquine as the first-line therapy against *P. falciparum* malaria (Figure 5B). As shown in a study on the Thai–Myanmar border,^10^ this can be explained by the long-acting effect of piperaquine compared with mefloquine, which may prevent and suppress *P. vivax* hypnozoite recurrences from liver during several weeks after the initial treatment. More recently, data from Ratanakiri Province (northern Cambodia) confirmed that recurrences occurred significantly later after dihydroartemisinin–piperaquine treatment (mean = 54.3 days, standard deviation [SD] = 3.5, range = 49–56) than after chloroquine treatment (45.7 days, SD = 8.1, range = 28–56) (*P* < 0.05) (Popovici and others, unpublished data). This may also explain why, despite the introduction of combo RDTs (detecting *P. falciparum* and non-*P. falciparum* malaria), *P. vivax* case counts did not significantly increase in Zone 1 the same way it was observed in the surrounding areas (Zones 2 and 3). Moreover, *P. vivax* prevalence declined slightly in all zones in 2012 after the introduction of dihydroartemisinin–piperaquine treatment in the entire country in 2010 and the mass distribution of LLINs in late 2011 and early 2012, suggesting that a portion of new, mosquito-borne infection *P. vivax* cases might be preventable by nets.

### Trends of *P. falciparum*:*P. vivax* ratio.

Overall, there has been a clear trend toward the reversal of the *P. falciparum*: *P. vivax* ratio over the past decade along with progressive reduction in malaria cases (Figure 4B). As reported by others, when *P. falciparum* is the predominant species, *P. vivax* tends to be obscured. As *P. falciparum* incidence declines, *P. vivax* appears to be more prominent than previously recognized. Some of the reasons are that *P. falciparum* is more easily recognizable under the microscope, and usually has higher parasitemia, so *P. vivax* tends to be missed. Moreover, relatively low *P. vivax* parasitemia may account for high rates of false-negative diagnoses by microscopy or RDTs, which are less sensitive for *P. vivax* than for *P. falciparum*,^11^ and many cases diagnosed as *P. falciparum* may actually be mixed *P. falciparum* and *P. vivax* infections. In addition, considering that malaria interventions in Cambodia do not particularly target *P. vivax* infections (primaquine treatment of radical cure is not used routinely), it is reasonable to assume that relapses are responsible for a large proportion of *P. vivax* cases.

### Distribution of age, sex, and occupation.

**Figure 6.**
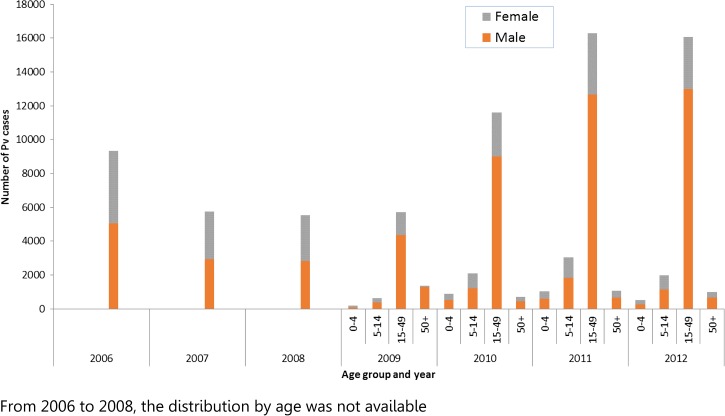
Distribution of the *Plasmodium vivax* cases detected in the public sector by age group and sex between 2006 and 2012, Cambodia.

Although there is a secular decline observed in the number of confirmed cases (all species) with a saw-tooth pattern of periodic increases observed in 2006 and 2009 (periodic variations in rainfall or climate change—La Niña; sudden changes in forest-related activities—military actions; illegal woodcutting; plantation development; sudden closure of factories and economic contraction, causing new migration and settlement in forests; extension of better diagnosis and referral services in remote areas; better coverage in regular reporting and increased reliability of reporting), the age group 15–49 years (both male and female) continues to predominate among *P. vivax* cases (Figure 6

). The male-to-female ratio of 1:1 was maintained from 2006 to 2008, but has changed to 3:1 in subsequent years, particularly among those 15–49 years of age. This is probably explained by the greater occupationally related mobility among men in this age group, thus placing them at a higher risk of exposure. Further analysis of age–sex distribution of cases in villages served by VMWs is also supportive of predominantly young and adult males (15–49 years of age) being the highest risk group. Overall, the same age–sex groups that get infected with *P. falciparum* also get infected with *P. vivax*.

### Recurrence of *P. vivax* infection.

A key biological difference between *P. vivax* and *P. falciparum*, with huge implications for vivax malaria control, is the occurrence of dormant *P. vivax* parasites in the liver (hypnozoites). After an infectious mosquito bite, vivax sporozoites migrate to the liver, invade hepatocytes, and then multiply, forming a schizont that eventually bursts, releasing merozoites into the bloodstream; these cells then invade the reticulocytes and enter the erythrocytic cycle. All *P. falciparum* liver-stage parasites undergo immediate asexual replication, whereas a proportion of *P. vivax* liver-stage parasites enter a dormant state (hypnozoite). Only a fraction of the initial sporozoites begin the first erythrocytic cycle inducing the primary episode of vivax malaria. The rest form hypnozoites that reactivate several weeks to months (or even years) after primary infection, causing multiple subsequent vivax malaria episodes called relapses. After the initial treatment of vivax malaria cases, recurrences can result from recrudescence (resistant parasites not killed by the initial treatment), reinfection (new infectious mosquito bite), or relapses (from dormant liver stages).

It is known that when patients with *P. falciparum* malaria are treated and followed for several weeks, a significant proportion will develop *P. vivax* malaria.^12^,^13^ *Plasmodium vivax* recurrences after falciparum malaria have been also observed in western Cambodia. In a combined analysis of 243 patients recruited in two malaria treatment trials, 20/43 (47%) of those with *P. falciparum* gametocytes on admission developed *P. vivax* malaria by day 28 of follow-up. The presence of *P. falciparum* gametocytes on an initial blood smear was associated with a 3.5-fold greater rate of vivax parasitemia posttreatment.^14^ Dedicated polymerase chain reaction (PCR) confirmed that only a low proportion of subjects (5/55, 9.1%) who developed vivax during follow-up had detectable *P. vivax* parasites in the peripheral blood at baseline. Molecular detection of *falciparum* gametocytes by reverse transcription PCR in a subset of patients strengthened the observed association, whereas PCR detection of *P. vivax* parasitemia at follow-up was similar to microscopy results. It has been suggested first that the presence of *P. falciparum* gametocytes may indicate increased malaria exposure in general: patients carrying gametocytes may be more likely to have acquired a *P. vivax* infection in the past and to harbor hypnozoites in their liver when they are infected with *P. falciparum* or second that the competition from a second malaria species (*P. vivax* in that case) may boost falciparum gametocytogenesis as an evolutionary adaptation. The authors of this study concluded that in Cambodia, the majority of *P. vivax* infections arising after treatment of falciparum malaria originate from relapsing liver-stage parasites.

Since 2012, the first-line treatment of uncomplicated malaria (all malaria species) recommended by the National Malaria Program is based on ACT treatment of 3 days (dihydroartemsinin–piperaquine). For vivax malaria, radical cure with primaquine (0.75 mg/kg weekly for 8 weeks or 0.25 mg/kg daily for 14 days) is recommended only if glucose-6-phosphate dehydrogenase (G6PD) deficiency testing is performed and G6PD deficiency is excluded. Practically, no point-of-care to detect G6PD deficiency is available in health facilities in Cambodia, and primaquine is not used routinely.^15^ This is a major problem for malaria elimination in Cambodia if access to radical cure with primaquine to eliminate hypnozoites continues to be limited.^16^

### Understanding *P. vivax* reservoir: data from national parasite prevalence surveys.

A series of large-scale malaria surveys (Cambodia Malaria Surveys [CMS]) conducted in 2004 (CMS2004, 3,363 households), 2007 (CMS2007, 2,923 households), 2010 (CMS2010, 3,802 households), and 2013 (CMS2013, 3,286 households) conducted by the CNM and Malaria Consortium, further supports the declines in malaria prevalence (all species) detected by both microscopic examination and PCR.^17^ These surveys, focused on populations at the highest risk determined by stratifying, first, by the level of risk of the provinces, and then by distance from the forests demonstrated that the prevalence of all *Plasmodium* infections detected by microscopy declined in each successive national survey since 2004. Weighted national prevalence by microscopy was 4.4% in 2004, 2.6% in 2007, and 0.9% in 2010, and had declined to 0.1% in 2013. In 2010 and 2011, a focused screening and treatment (FSAT) using PCR for case detection was carried out in villages of Pailin Province, the artemisinin resistance hotspots, supported this observation.^18^ It had been anticipated that a large number of asymptomatic cases would be discovered. In fact, the FSAT 2010 screened 6,931 people and found only 0.86% *P. falciparum* positive and 1.04% *P. vivax* positive; the FSAT 2011 screened 5,229 people and found only 0.11% *P. falciparum*–positive cases and 0.52% *P. vivax*-positive cases. These consecutive surveys confirmed *P. vivax* to be the more common species in that area, albeit at a very low prevalence. This observation was also confirmed in Preah Vihear Province (located in northeastern Cambodia, along the Thailand and the Lao People's Democratic Republic borders) in 2013.^19^ The objective of the study was to compare the diagnostic accuracy of a malaria PCR assay, from dried blood spots (5 μL of blood) and different volumes of venous blood (50 μL, 200 μL, and 1 mL). On a set of 521 field samples collected in two different transmission areas in northern Cambodia, no significant difference in the proportion of parasite carriers, regardless of the volume used, was observed. However, the authors found that the 5-μL dried blood spot method missed 27% of the samples detected by the 1-mL venous blood method, and most of the missed parasite carriers (84%) were infected by *P. vivax* with a parasite density below 100 parasites per milliliter. More recently, Tripura and others^20^ followed for 1 year at 3-month intervals the entire population of three Cambodian villages in Pailin Province and tested each villager by ultrasensitive quantitative PCR to detect malaria parasites. In the initial cross-sectional survey (M0), they found that among 1,447 asymptomatic residents, 32 (2.2%) were *P. falciparum* carriers, 48 (3.3%) *P. vivax* carriers, and four (0.3%) mixed infections carriers. In 142/1,447 (9.8%) cases, they were not able to identify the species. Monthly follow-up without treatment of 24 adult participants with asymptomatic mono or mixed *P. falciparum* infections revealed that 3/24 (13%) remained parasitemic for 2–4 months, whereas the remaining 21/24 (87%) participants had cleared their parasitemia after 1 month. In contrast, 12/34 (35%) adult participants with *P. vivax* monoinfection at M0 had malaria parasites (*P. vivax* or unindentified *Plasmodium* sp.) during four or more of the following 11-month surveys. Their data showed limited duration of *P. falciparum* carriage, but prolonged carriage of *P. vivax* infections, suggesting that radical treatment of *P. vivax* infections by 8-aminoquinoline regimens may be required to eliminate all malaria from Cambodia.

### *Plasmodium vivax* transmission: entomological background.

Most of information described in this paragraph is derived from *P. falciparum* vector studies, as very little data are available about *P. vivax* transmission in Cambodia. The main malaria transmission season in the country typically runs from June to October in parallel with the rainy season. The common vectors prefer forested areas, so the risk of infection is linked to proximity to forests, affecting particularly migrant populations, mobile populations working in or close to forests, and forest goers.^21^ Twenty-five malaria vector species have been identified in Cambodia between 2007 and 2013.^22^ *Anopheles* *maculatus* s.l. is present throughout Cambodia, *Anopheles minimus* s.l. more prevalent in the west, and *Anopheles dirus* in the northeast. Other vectors such as *Anopheles barbirostris*, *Anopheles philippinensis*, *Anopheles vagus*, and *Anopheles hyrcanus* are also present in Cambodia.^23^–^25^ *Anopheles dirus* is found in the forested mountains and foothills, cultivated forests, and rubber plantations, whereas *An. minimus* is frequent outside the forests or in areas where the forests have been cleared, breeds along grassy streams in clear, unpolluted water, and its abundance is determined by water velocity and types of vegetation.^25^ *Anopheles maculatus* is found in hilly or mountainous areas and breeds in or near permanent or semipermanent bodies of clean water like streams or rivers. *Anopheles epiroticus* is able to breed in water with some salinity, and is therefore typically found in Cambodia's coastal areas. These vectors bite during all hours of the evening, but peak biting hours are usually found to be between 8 pm and 12 am.^22^

Malaria incidence in Cambodia is changing rapidly due to environmental and socioeconomic changes coupled with effective interventions.^21^ Deforestation (through both legal concessions granted by the government as well as illegal logging) has significantly impacted the landscapes with plantations of rubber, palm oil, and fruit orchards replacing the jungles and altered the scenarios of vector-borne diseases including malaria. In neighboring Thailand, *An. dirus* and *An. maculatus* have been found to adapt to varying environmental conditions despite the preference of natural forest habitats.^26^ In Thailand, *An. dirus* were found in areas where natural forests were replaced with orchards, tea, coffee, and rubber plantations.^27^,^28^ This species is notoriously difficult to control due to its exophilic behavior, scattered breeding sites, and late biting times. As secondary vectors are often less anthropophilic, and might be more exophagic and early biting, planning of vector control should also take into account their behaviors.^21^ Moreover, secondary vectors could be better vectors for *P. vivax* than for *P. falciparum*, as the extrinsic incubation period of *P. vivax* is shorter.

There is no available entomology data on *P. vivax*–specific vectors in Cambodia. All main malaria vectors mentioned above were known to be more abundant for transmitting *P. falciparum* and less abundant for *P. vivax*. A recent study to identify anopheline vectors in relation to malaria transmission and disease risk in Pailin and Pursat, western Cambodia, was conducted in August–September and November–December 2005.^29^ Specimens (*N* = 16,160) of seven anopheline species obtained by human landing collections were tested by PCR for *P. falciparum* and *P. vivax*, it was found that 11 *An. dirus* of 9,233 *Anopheles* (*An. dirus*, *An. minimus*, *An. Maculatus*, *An. barbirostris*, and *Anopheles jamesii*) were positive for *P. falciparum* and four *An. dirus* and one *An. minimus* were positive for *P. vivax*.^29^ It is believed that all main malaria vectors such as *An. dirus*, *An. minimus*, *An. maculatus*, and *An. barbirostris* could transmit both *P. falciparum* and *P. vivax*. Considering the increasing changes in their habitats (more plantations and rural development projects), dominant vector species in Cambodia may change, which may or not affect transmission of malaria parasite species.^30^ The exophilic behavior and early biting habits observed in some endemic areas are threatening the effectiveness of the most common vector control measures such as IRS and LLINs. This issue of “residual transmission” is being recognized in Cambodia.^21^ In some settings elsewhere, 50% of infective bites took place before sleeping time and daytime biting in shaded jungle areas have been noted.^31^ Therefore, even for very low and focused malaria transmission such as in Cambodia, the role of outdoor feeding, outdoor resting, and/or zoophilic vectors in sustaining malaria transmission needs to be carefully taken into consideration for the control of both *P. falciparum* and *P. vivax*.

#### Radical cure with primaquine and G6PD deficiency issue.

The only licensed drug available for the elimination of hypnozoites is primaquine. In combination with a drug for the treatment of blood-stage infection, it provides the only treatment strategy for the radical cure of vivax malaria. However, the safety of primaquine use remains a serious concern. When patients with severe (for males) or mild (for heterogeneous females) defects of G6PD activity receive primaquine, this drug may cause dose-dependent hemolysis resulting in life-threatening acute hemolytic anemia requiring careful medical monitoring and possible blood transfusion. G6PD deficiency in Cambodia is predominantly of the Viangchang variant (871G > A) which leads to severe deficiency. Studies have shown 10–15% prevalence of this variant in the Khmer populations, particularly males.^32^–^34^ This differs from Thailand, where the milder deficiency variant, Mahidol, is more common. From September 2010 to 2012, a 2-year survey of G6PD deficiency and hemoglobinopathies assessed by quantitative enzyme activity assay and hemoglobin electrophoresis, respectively, was conducted in malaria patients presenting to 19 health centers in all over Cambodia. A total of 2,408 confirmed malaria cases by microscopy of mean age 26.7 (range = 2–81) years were recruited from mostly western Cambodia (71.9%); males outnumbered females at the proportion of 3.9:1. *Plasmodium falciparum* was present in 1,443 (59.9%) and *P. vivax* in 965 (40.1%) patients. Mean G6PD activity was 11.6 (95% confidence interval = 11.4–11.8) U/g Hb. G6PD deficiency was present in 13.9% of all malaria patients (335/2,408), and severe deficiency (including World Health Organization [WHO] Class I and II variants) was more common in western (9.1%) than eastern (5.1%) Cambodia (*P* = 0.01). The researchers found that G6PD deficiency (as well as anemia and hemoglobinopathies) were common in malaria patients, and that decision for a large-scale deployment of primaquine in Cambodia should be preceded by primaquine safety studies in parallel with field evaluation of rapid, point-of-care G6PD tests.^35^

In Cambodia, primaquine had not been mentioned in the national malaria treatment guidelines until May 2012. Primaquine is not used in Cambodia because the entire medical community heeds a common wisdom that primaquine use is dangerous if given to severe G6PD-deficient individuals.^16^ Because primaquine is not prescribed in routine for radical treatment of *P. vivax*, and no attempt is usually made to determine past history of infection, most *P. vivax* cases naturally develop multiple relapses, and each episode of relapse is recorded as a new case. If primaquine radical treatment was, it would likely lead to a substantial reduction of *P. vivax* cases. Development of reliable, easy-to-use RDTs to detect G6PD deficiency at point-of-care is essential to deploying primaquine therapies. A new tool, the CareStart^™^ G6PD deficiency screening test (AccessBio, Somerset, NJ), has been assessed recently in Cambodia by comparing its performance to quantitative G6PD enzyme activity using a standardized spectrophotometric method (“gold standard”).^32^ Blood samples (*N* = 903) were collected from Cambodian adults living in Pailin Province, western Cambodia. G6PD enzyme activities ranged from 0 to 20.5 U/g Hb (median = 12.0 U/g Hg). On the basis of a normal hemoglobin concentration and wild-type G6PD gene, the normal values of G6PD enzymatic activity for this population were 5.5–17.2 U/g Hg (95th percentile). Ninety-seven subjects (10.7%) had 3.6 U/g Hg and were classified as G6PD deficient. Prevalence of deficiency was 15.0% (64/425) among men and 6.9% (33/478) among women. Genotyping was done in 66 G6PD-deficient subjects, and 63 of these exhibited findings consistent with the Viangchang variant (871G > A). The sensitivity of CareStartG6PD deficiency screening test prototype was disappointing (68%). More recently, the third generation of the CareStart G6PD RDT (www.accessbio.net) was found to be more capable and reliably detected moderate and severe G6PD-deficient individuals (enzyme activity < 30%), suggesting that this novel point-of-care is a promising tool for tailoring appropriate primaquine treatment of malaria elimination by excluding individuals with severe G6PDd for primaquine treatment.^34^

### *Plasmodium vivax* drug resistance.

Therapeutic efficacy studies (TES) using WHO protocol have been carried out at sentinel sites around Cambodia since 1991.^36^ In recent years, TES data were also available for *P. vivax*, in addition to *P. falciparum*.^37^ Currently, dihydroartemisinin–piperaquine recommended as the first-line drug for both uncomplicated *P. falciparum* and *P. vivax* infections in Cambodia, continues to be fully effective against *P. vivax*, whereas dihydroartemisinin–piperaquine failure for *P. falciparum* treatment is emerging in some areas in western and northern Cambodia.^38^–^43^

Sporadic and isolated failures of chloroquine against *P. vivax* were detected between 2003 and 2005 in Sampouvloun (Battambang Province, western Cambodia) as a part of antimalarial drug efficacy monitoring by the national control program. The last TES conducted at four sites (two in the west in Pailin and Veal Veng districts and two in the east, in Rovieng and Veun Sai districts) from 2008 to 2011 (day 28 follow-up, PCR uncorrected, WHO protocol) showed proportions of treatment failure of 17.4% in Ratanakiri Province, 2010, following a chloroquine regimen, whereas in the same areas, dihydroartemisinin plus piperaquine treatment was 100% effective.^39^ However, it is worth noting that chloroquine resistance in this area was not fully confirmed due to the lack of chloroquine blood concentration measurement and genotyping data (between the isolates on day 0 and those on the day of recrudescence). In contrast to drug-resistant *P. falciparum*, which is more common in the western provinces, *P. vivax* “suspected” resistance to chloroquine was detected in the eastern part of Cambodia,^39^ whereas chloroquine is still effective in western Cambodia.^44^ On the basis of these data, it was decided in 2012 to use dihydroartemisinin–piperaquine as the first-line therapy for uncomplicated *P. vivax* malaria, mainly to simplify the management of the malaria cases, especially at community level.

In search of an alternative ACT regimen, a phase III randomized, double-blind clinical trial of pyronaridine–artesunate combination was conducted at five centers across Cambodia, Thailand, India, and Indonesia.^45^ Patients with microscopically confirmed *P. vivax* monoinfection were randomized (1:1) to receive pyronaridine–artesunate (target dose of 7.2:2.4 to 13.8:4.6 mg/kg) or chloroquine (standard dose) once daily for 3 days. Each treatment group included 228 randomized patients. The study concluded that pyronaridine–artesunate efficacy in acute uncomplicated *P. vivax* malaria was at least as good as that of chloroquine in Cambodia.

## Current Malaria Control Activities and Interventional Measures

For several years, the CNM has decentralized implementation of malaria control activities to the provinces, operational districts (ODs), and peripheral health facility levels to be in line with the government's decentralization policy. Currently, there is no *P. vivax*–focused strategy and financing despite the fact that *P. vivax* is the key species the national control program will face with from now on. Experiences from elsewhere indicated that eliminating *P. vivax* is more difficult and usually requires a prolonged process. Therefore, efforts are made to mobilize additional resources required to embark on the elimination strategy and ensure further strengthening of partnerships with key donors and stakeholders at different levels.

At present, the National Strategic Plan for Elimination of Malaria proposes a sequential progression toward preelimination and elimination. Among the activities being prioritized in the move toward malaria elimination are: universal LLIN coverage, universal access to free-of-charge antimalarials (e.g., via VMW scale-up), case confirmation by slide/RDT or PCR, health and malaria information system strengthening, active case detection, case investigation and response, linkages with the private sector for diagnosis, treatment and reporting, monitoring of drug resistance and parasite genotyping, strong border control and improved screening tools, innovative protection of night-time outdoor workers, targeting mobile populations, and so on.

Some interventions are also planned specifically for *P. vivax* such as G6PD testing before administration of primaquine and access to primaquine for radical treatment of *P. vivax* malaria, but no agenda has been defined. In addition, there has been increased emphasis on surveillance in recent years. Full investigation of every malaria case and all malaria-related deaths is being encouraged in areas that are approaching elimination/preelimination.

### Case management.

Cambodia follows current WHO guidelines on Test, Treat, Track for people living in endemic areas and will expand the efforts to all mobile/migrant populations at risk. VMWs will be scaled up to cover 4,528 villages (instead of 2,539) by 2020, thereby ensuring universal access to early diagnosis and treatment. When primaquine will be widely deployed for the radical cure of *P. vivax*, it is envisaged that VMWs will play a role in documenting compliance to the 14-day regimen and following up *P. vivax* cases for 28 days to observe treatment outcomes.

### Vector control strategies and coverage.

Standard vector control strategy uses LLINs and hammock nets distributed in mass campaigns at 3-year intervals to all people at risk of malaria, defined as people living within 2 km of the forest. The private sector involving rubber plantations, palm oil plantations, fruit orchards, mining, construction, hydroelectric power projects, and so on will be put under expanding bed net coverage (i.e., 90% coverage LLINs among all at-risk populations). Personal protection has recently been researched^46^ and the analysis of data is still in progress. For instance, results regarding the impact of repellent use by villagers in Cambodia, once available, will help guide the implementation of personal protective measures (Sluydts, unpublished data).

### Health communication.

Health communication is being promoted through behavior change communication and information, education, and communication strategies in addition to standard case management and vector control approaches. Innovative measures to reach mobile, migrant, and other hard-to-reach populations are also used to distribute bed nets, to spread messages on malaria prevention and care through taxi drivers.^47^

### Surveillance system, monitoring, and evaluation.

Before 2009, the main source of malaria case data in Cambodia came from the national Health Information System, which provided aggregate data at OD level. Although useful for reporting total cases, these data were not sufficient to support the stratification of malaria risk by villages. Malaria risk stratification of villages had been based on distance from the forest but with outdated maps. In 2009, as a part of artemisinin-resistant containment, a malaria information system (MIS) was put in place for more efficient and timely reporting process and feedback. The system improved the overall efficiency of the program operation and fulfilled the need for 1) demographic data of all at-risk villages for planning interventions, 2) data relating to bed net distribution and treatment, 3) monthly malaria data by species at village level to identify villages with high incidence and possible transmission, 4) data relating to stock-outs of essential malaria drugs and supplies, and 5) real-time data on individual *P. falciparum* patients who are still blood-smear positive 3 days after initiation of treatment (an early signal of possible artemisinin resistance). All these data were disaggregated by species thus allowing for the analyses of *P. vivax*–specific trends.

In addition, the following data items were identified as being required at a later stage of the program as it moved toward elimination: 1) malaria data relating to private sector outlets, 2) real-time data of all *P. falciparum* (and ultimately *P. vivax*) cases, and 3) real-time data about malaria outbreaks in high-transmission settings. The MIS now allows for village-level malaria stratification, which is being used for refining deployment of malaria control and preelimination activities.^48^ The MIS also has comprehensive and real-time reporting and geospatial analytical applications that allows for exporting of raw data and mapping of data using Google Earth. Further scaling up and refinement of this system will support the national control program to move toward elimination phase allowing prompt response to every *P. falciparum* case (and ultimately every *P. vivax* case). Currently, no intervention is targeting *P. vivax* specifically. However, when primaquine radical treatment will be rolled out countrywide in conjunction with point-of-care G6PD testing, adaptation of these systems will be needed to monitor *P. vivax* trends and impact of the primaquine intervention more closely.

## Constraints and Obstacles

In recent years, procurement delay has largely been responsible for a lag between what is planned under the national control program and what have actually been implemented. For example, delays in the procurement of RDTs and ACTs have resulted in periodic episodes of stock-outs of diagnostics and antimalarials in the country. Disbursement delays, mainly from the GFATM, have also affected implementation at field level, for example, lack of cash flows for payment of VMW incentives prohibited VMW meetings scheduled monthly, when all VMW records were to be verified and RDT/ACT stocks were to be replenished.

Cross-border issues, conflict areas, migrant populations, remote villages, poor drug quality, and counterfeit drugs remain key challenges for malaria control in Cambodia regardless of parasite species. As a consequence of the operation to contain artemisinin resistance, the first phase of malaria elimination in Cambodia aims at eliminating *P. falciparum*. In the second phase, however, *P. vivax* is likely to pose a major challenge. The need for more efforts and longer time to eliminate *P. vivax* must be planned ahead and budgeted for while priority is set to keep vigilance against potential reemergence of *P. falciparum* infections including from neighboring countries.

Special attention will have to be given to a number of implementation challenges specifically relevant to *P. vivax* elimination: 1) maintaining maximal intervention efforts for an extended period to eliminate indigenous infections, 2) ensuring the safety of primaquine and compliance with its prolonged treatment course, 3) guarding against the risk of reintroduction of *P. vivax* into areas without transmission, but where competent anopheline vectors remain, through the entry of *P. vivax* carriers, and 4) strengthening and maintaining adequate malaria surveillance beyond elimination to prevent reintroduction.

## Conclusions

Cambodia is a good example of malaria-endemic countries with an impressive decline in malaria burden in the past decade, with a slower decline of *P. vivax* compared with *P. falciparum*. Drug resistance has been the major obstacle for malaria control in Cambodia, often regarded as the epicenter of multidrug *P. falciparum*–resistant malaria. Rigorous control efforts, primarily targeting artemisinin-resistant *P. falciparum* from 2009, have made significant impacts to reduce malaria cases and deaths to the lowest levels ever. Like many *P. falciparum*/*P. vivax* coendemic countries in Asia and South America, as *P. falciparum* cases drop, the burden of *P. vivax* becomes more prominent. The extent of problems associated with *P. vivax*, including its overall health and socioeconomic impacts, might not have been anticipated before. Eliminating *P. vivax* is known to require a lengthier process than eliminating *P. falciparum*. Without strengthening malaria control policy to target *P. vivax* and to seriously implement specific actions against the parasite, especially maximizing access to radical cure, it would be unlikely for Cambodia to achieve the ambitious goal of malaria elimination by 2025.
